# Impact of the Autophagy Machinery on Hepatitis C Virus Infection

**DOI:** 10.3390/v3081342

**Published:** 2011-08-04

**Authors:** Marlène Dreux, Francis V. Chisari

**Affiliations:** 1 Department of Immunology and Microbial Science, The Scripps Research Institute, La Jolla, CA 92037, USA; 2 Ecole Normale Supérieure de Lyon, Lyon, F-69007, France; 3 Université de Lyon, Lyon, F-69007, France; 4 INSERM, U758, Lyon, F-69007, France

**Keywords:** hepatitis C virus, HCV, autophagy, autophagic vesicle, autolysosome, unfolded protein response, ER-stress response, innate host response, interferon, RIG-I

## Abstract

Autophagy is a cellular process that catabolizes cytoplasmic components and maintains energy homeostasis. As a stress response, the autophagy machinery interconnects a wide range of cellular pathways, enhancing the spread of certain pathogens while limiting others, and has become a highly active research area over the past several years. Independent laboratories have recently reported that autophagy vesicles accumulate in hepatitis C virus (HCV) infected cells and that autophagy proteins can function as proviral factors required for HCV replication. In this review, we summarize what is currently known about the interplay between autophagy and HCV and the possible mechanisms whereby autophagy proteins might favor HCV propagation.

## 1. Introduction

Autophagy is a homeostatic process by which cells break down and recycle cytoplasmic organelles and protein aggregates that are too large to be degraded by the proteasome. During autophagy, cytoplasmic components are sequestered by a membrane, called a phagophore or isolation membrane, that expands to form double-membrane vesicles termed autophagosomes. Autophagosomes can fuse with vesicles of the endocytic pathway to form amphisomes that eventually fuse with lysosomes where the sequestered material is degraded. The core molecular machinery of autophagy—the autophagy (ATG) proteins—orchestrate this dynamic membrane rearrangement [1]. Our current understanding of the molecular regulation of the autophagy pathway has been described in detail in other reviews (e.g., see [1–5]). The autophagy machinery is thought to have evolved as a stress response that permits unicellular eukaryotic organisms to survive, probably by regulating energy availability and organelle quality. The same autophagy machinery has diversified functions in higher eukaryotic organisms so as to regulate multiple defensive responses to various forms of stress. It notably plays an essential role in the removal of potentially pathogenic protein aggregates from the cell during aging or degenerative processes. Not surprisingly, the autophagy machinery can also degrade components of intracellular microorganisms, including viruses, bacteria and protozoa (reviewed in [6]). Additionally, the autophagy machinery can play a pivotal role in the host response against these infections. Autophagy proteins serve to balance the induction and suppression of antiviral and inflammatory responses, thereby regulating the protective and potentially pathogenic consequences of these responses (reviewed in [7]). Autophagy is activated by cellular stress, including that induced by viral infection. However, certain viruses can antagonize its initiation as well as its maturation (reviewed in [6]). This complex reciprocal relationship between viruses and the autophagy machinery may influence the severity and the outcome of an infection, including whether it resolves or persists (reviewed in [6]).

The hepatitis C virus (HCV) is a major causative agent of acute and chronic liver diseases worldwide. 130–170 million people are persistently infected with HCV and have a high risk of developing liver cirrhosis and hepatocellular carcinoma [8]. A hallmark of HCV is its high propensity to persist with up to 80–90% of infected individuals failing to eliminate the virus. A prophylactic vaccine is not available and its development has been seriously hampered by the high genetic variability of the virus. Treatment options for chronic HCV infection are also limited. Indeed the current therapy, a combination of polyethylene glycol-conjugated interferon-α and ribavirin, is associated with numerous side effects, has many contraindications and on average only approximately 50% of those patients who can qualify for treatment, tolerate its toxicity, and complete six months of therapy are cured [9]. A large number of direct-acting antiviral agents are under development at this time with the hope of eventually replacing interferon and ribavirin as the treatment of choice for chronic HCV infection.

HCV, a single stranded positive sense RNA virus, is a member of the *Flaviviridae* family. Its 9.6-kb RNA genome encodes a long open reading frame that is co- and post-translationally cleaved by cellular and viral proteases (reviewed in [10]) into structural proteins (core, E1, E2) that constitute the major viral components of infectious particles, and non-structural proteins (p7, NS2, NS3, NS4A, NS4B, NS5A and NS5B) that are required at multiple levels of the virus life cycle including viral RNA replication [11] and infectious particle assembly [12]. The single open reading frame is located between two untranslated regions (UTR) that contain RNA sequences essential for RNA translation and replication [13–15]. HCV infection is initiated by binding of enveloped viral particles to cell surface receptors, followed by their internalization into endosomes in a clathrin-dependent manner (reviewed in [16,17]). Release of the plus-strand RNA viral genome into the cytoplasm is thought to occur after low pH-induced fusion of the viral and endosomal membranes, a process that is mediated by the viral envelope glycoproteins [16,18,19]. Translation of the plus-stranded viral RNA genome is driven by an internal ribosomal entry site (IRES) sequence present within the 5′ UTR [10]. Subsequent cleavage of the polyprotein into individual viral proteins leads to establishment of replication complexes in ER-derived membranous compartments where viral RNA replication occurs via minus strand viral RNA synthesis [10]. Progeny viral genomes are either translated to produce additional viral proteins or packaged to assemble progeny infectious virus. Viral particle assembly is thought to occur in an ER-derived compartment in close proximity to lipid droplets (reviewed in [12,20,21]). The HCV assembly process is dependent not only on structural and non-structural proteins (p7, NS2, NS3 and NS5A) [22–32] but also on many cellular factors including those involved in lipoprotein biosynthesis [33–40].

Recently, several papers have been published that shed light on the ability of HCV to modulate the autophagy pathway and on the capacity of several autophagy proteins to positively regulate HCV productive infection [41–46]. Here we review these recent advances, present our understanding of the mechanism(s) by which autophagy proteins can enhance HCV infection, and discuss the implications of those concepts for the survival of HCV in infected cells.

## 2. HCV Regulates the Autophagy Pathway

A broad spectrum of DNA and RNA viruses induce the accumulation of autophagosomes or autophagy-related vesicles in infected cells [41,42,46–69]. Ait-Goughoulte and colleagues initially showed that serial passage of an HCV genotype 1a isolate (H77) in immortalized human hepatocytes provokes accumulation of microtubule-associated protein 1 light chain 3 β (LC3B)-positive vesicles, a characteristic feature of autophagic vesicles [59]. The occurrence of increased numbers of autophagy vesicles in HCV infected cells has been confirmed and extended to other cell types and viral strains using different detection methods [41–44,46,68]. This phenotype does not require HCV cell entry nor the production of infectious virus, since human hepatoma-derived cell lines containing a replication-competent subgenomic HCV genome that cannot produce infectious virus particles display the characteristic features of autophagic vesicle accumulation [46,68]. Furthermore, since degradation of long-lived proteins and p62/sequestosome 1, a polyubiquitin binding protein known to be degraded via autophagy [70], were not enhanced in HCV replicating cells, Sir *et al.*, proposed that autophagy seems incomplete in such cells [46]. In contrast, Ke and Chen demonstrated that autophagic vesicle maturation and autolysosome formation occur in HCV infected cells [44]. This apparent discrepancy might reflect differences in the kinetics and magnitude of HCV replication and protein expression in the conflicting experiments [44,46] or the possibility that infected cells might contain specific subsets of autophagic vesicles that engulf and degrade only a restricted set of cellular proteins [7]. Additional studies are clearly needed to resolve this uncertainty.

## 3. The Unfolded Protein Response Induces Autophagy

Several viruses have been reported to induce a cellular ER stress response, also termed the unfolded protein response (UPR) [71], that, in turn, can induce autophagy [4,44,46,72–75]. The UPR might be triggered by viral polyprotein synthesis or viral replication in the ER or ER-derived structures [71]. The UPR response allows infected cells to recover from ER stress by attenuating translation and up-regulating the expression of chaperone proteins and degradation factors that refold or eliminate misfolded proteins [71]. Several independent studies suggest that HCV induces the UPR *in vitro* [44,46,76,77] and that it is detectable in liver biopsies of HCV infected patients [78] but the molecular mechanism(s) responsible for UPR induction by HCV is unclear. For example, the isolated expression of NS4B, a viral protein involved in formation of the HCV replication complex [79–81], induces the UPR via transactivation of ATF6 and IRE1-XBP1 [76,82]. Alternatively, the viral E2 glycoprotein possesses a retention motif in its transmembrane domain that, in complex with the viral E1 glycoprotein, mainly resides in the ER [83] where it could form aggregates that contribute to ER stress activation [84]. Consistent with previous observations [4,72–75], down-regulation of a variety of UPR modulators (e.g. PERK, IRE1α, CHOP and ATF6) inhibits HCV induced LC3-phosphatidylethanolamine conjugation, a hallmark of autophagic vesicle accumulation [44,46]. By analogy to the proposed mechanism of autophagy induction by hypoxia-triggered UPR, one can speculate that HCV-induced enhancement of CHOP expression [44] might activate autophagy by up-regulating Atg5 and LC3B expression [74]. Alternatively, it is possible that HCV-induced eIF2α phosphorylation via PERK [46], in turn, may activate autophagy as previously reported [69,73]. Nonetheless HCV replication and HCV protein accumulation are reduced in cells in which UPR proteins are down-regulated [44,46]. Thus additional studies will be required to determine if HCV protein(s) or HCV RNA modulate the autophagy pathway directly, or indirectly by activating the UPR.

## 4. The Autophagy Machinery Positively Regulates HCV Replication

Several independent laboratories have reported that autophagy proteins, *i.e.*, Beclin 1, LC3, Atg4B, Atg5, Atg7 and Atg12, are required for productive HCV infection [41–45]. Dissection of the individual steps of the HCV life cycle suggests that these proteins specifically modulate the onset of translation of incoming HCV RNA and, therefore, the establishment of HCV replication [41], an observation further confirmed by independent studies [43,44]. Additionally, Tanida *et al*. observed that the release of HCV core and infectious particles from infected cells is reduced in Beclin 1- and Atg7-down-regulated cells, and they proposed that, in addition to facilitating the initiation of viral replication, autophagy proteins also contribute to HCV particle assembly and/or egress [42].

## 5. Regulation of Innate Immune Signaling by Autophagy Proteins

Recognition of viral RNA by innate response receptors triggers the production of antiviral molecules, e.g., type 1 interferon (IFN-α and β) and inflammatory cytokines [85] that are critical to control infection and to potentiate immune responses. Viral RNAs are recognized by cytosolic receptors (e.g., RIG-I, MDA5, NLRPs) and by toll-like receptors (TLRs) present in endosomal/lysosomal compartments of the cell [85]. Importantly, autophagy proteins are emerging as key regulators of the innate host response against viral infection by either activating or inactivating the induction of antiviral molecules in infected cells [6,86–91]. It is possible, therefore, that autophagy proteins can regulate the balance between the restriction of viral propagation and the pathogenetic potential of an overly strong host response (reviewed in [7]). For example, Atg5 represses RIG-I-triggered IFNα/β production in vesicular stomatitis virus (VSV)-infected cells [89,90]. Indeed, Journai *et al*. showed that Atg5-Atg12 conjugates interact with RIG-I and IFNβ promoter stimulator 1 (IPS-1) (also called mitochondrial antiviral signaling proteins (MAVS), virus-induced signaling adaptor (VISA) or CARD adaptor-inducing IFNβ (Cardif)), a downstream partner of RIG-I that transmits signaling via interaction of their caspase recruitment domains (CARDs) [89]. The authors propose that the Atg5-Atg12 conjugate intercalates between the RIG-I and IPS-1 CARD domains and inhibits signal transduction, thereby suppressing type I IFN production [89]. Other observations suggest that autophagy limits RIG-I-induced type I IFN promoter activation via the clearance of dysfunctional mitochondria and the attendant reduction in reactive oxygen species (ROS) production [90]. Additionally, Soucy-Faulkner *et al.* demonstrated that NOX2, an NADPH oxidase (NOX) enzyme that liberates cellular ROS, positively controls RIG-I-mediated signaling by regulating MAVS expression [92]. Consistent with these previous reports, Ke and Chen showed that down-regulation of Atg5 markedly increased IFNβ promoter activation triggered by overexpression of a constitutively active RIG-I mutant *in vitro* [44]. IFNβ and ISG expression induced by transfection of the HCV-PAMP RNA, consisting of a homopolymeric uridine and cytidine (poly-U/UC) motif in the 3′ UTR of the HCV genome that has been identified as a major RIG-I-ligand [93,94], was also greatly potentiated in Atg5 and LC3 knockdown cells [44]. Using immortalized human hepatocytes, Shrivastava *et al.* also proposed that Beclin 1 and Atg7 decreased IFNβ and ISG expression [45]. Interestingly, using lysosome-associated membrane protein 2 (LAMP2)- and Rab7-down-regulated cells, Ke and Chen demonstrated that autolysosome maturation is required to repress HCV-PAMP-induced RIG-I signaling [44]. Moreover, UPR modulators (*i.e.*, CHOP, IRE1α, ATF6 and PERK) limit RIG-I-mediated induced signaling, and chemical induction of the UPR and autophagy reciprocally decrease this signaling [44]. Since similar results were obtained with RIG-I-mediated signaling triggered by a Dengue virus genome-derived poly-U sequence [44], collectively, these results suggest that autophagy and UPR regulators play an important role in suppressing antiviral signaling induced by PAMPs derived from members of the *flaviviridae* [44]. Based on these results and previous studies demonstrating that autophagy proteins limit VSV-induced IFN signaling, it appears that autophagy-mediated repression of IFNβ and ISG expression extends to a broad spectrum of RIG-I ligands, in multiple cell types, and host species [89,90].

Nonetheless, HCV aborts the RIG-I-induced innate immune response by NS3/4A-dependent proteolysis of IPS-1 [95–98] which, together with a currently undefined NS3/4A-independent inhibitory mechanism [99], blocks the induction of IFNβ and IFN stimulated genes (ISGs) in HCV-infected cells [99,100]. In agreement with this observation, Ke and Chen showed that HCV infection itself (*i.e.*, in the absence of RIG-I overexpression and HCV PAMP transfection) does not trigger IFNβ and ISG expression, even in autophagy protein-deficient cells [44]. Additionally, autophagy proteins are also required for HCV propagation in Huh-7.5.1 cells that are deficient for RIG-I signaling [101]. Thus, the extent to which amplification of RIG-I signaling by autophagy proteins accounts for reduced HCV replication in autophagy protein-deficient cells remains unclear at present, perhaps suggesting that autophagy proteins control HCV replication by RIG-I-independent mechanisms as well.

## 6. Dynamic Membrane Remodeling by the Autophagy Machinery

As a dynamic membranous process, autophagy has been proposed to produce a scaffold for intracellular membrane-associated, replication factories for RNA viruses that, like HCV, are known to replicate and assemble on cytoplasmic membranes. Evidence suggests that some viral RNA replication/transcription complexes, e.g., for nidoviruses and rotaviruses, are anchored on double membrane vesicles (DMVs) that resemble autophagosomes and are decorated with LC3 [64,102–111]. In contrast, confocal microscopic analysis of HCV-infected cells provides little evidence of colocalisation of LC3 and viral proteins [41,42,46,59]. Nonetheless, by discontinuous sucrose gradient analysis, Ferraris *et al.* detected DMVs in fractions containing HCV RNA and proteins that apparently co-sedimented with LC3 [68]. Interestingly, they further showed by immuno-EM analysis of HCV subgenomic replicon cells, that some DMVs contain the HCV NS5A protein [68]. These observations suggest that, in HCV replicating cells, some viral elements may be present in structures resembling autophagic vesicles [68]. Additionally, Guevin *et al*. reported, in a heterologous yeast system, that the HCV NS5B RNA-dependent RNA polymerase interacts with ATG5 [43] via a highly conserved patch of basic amino acids at its C-terminus [112]. Moreover, they detected strong colocalization between NS5B and ATG5 that is temporally restricted to early time points of infection [43], suggesting that NS5B/ATG5 interaction could be necessary for the establishment of HCV replication but not to maintain it. This is consistent with earlier observations from our laboratory that key regulators of autophagic vesicle formation are required to initiate translation and replication of incoming viral HCV RNA in *de novo* infected cells, but they are not required to maintain this process once viral replication is established [41]. Collectively, these results suggest that the host factors required for translation of the incoming viral RNA are distinct from those necessary for translation and replication of progeny HCV RNA and that some of those host factors are autophagy proteins [41,113].

The origin of the autophagosomal membrane is still a pending question. Nonetheless, several recent studies suggest that the ER is crucial for autophagosome formation, since autophagy proteins are recruited to certain domains of the ER or to closely attached structures and direct connections between the ER and autophagosomal membranes have been demonstrated [114–120]. By remodeling ER membranes, autophagy proteins or autophagic vesicles might provide an initial membranous support for translation and replication of incoming RNA, prior to accumulation of viral proteins and before the eventual establishment of HCV-induced cellular modifications [10]. Alternatively, it is possible that autophagy proteins contribute to the cytoplasmic transport of the incoming viral RNA to the translation apparatus, a hitherto unstudied step of HCV infection that, by its unique nature, could be different for incoming RNA *vs*. progeny viral RNA produced during viral replication. Future work will be needed to better understand this interplay. Nonetheless, it is conceivable that a restricted subset of autophagy markers may localize transiently at the site of HCV translation and replication. In support of this concept, only non-lipidated-LC3 has been detected at the site of mouse hepatitis virus (MHV) replication [111]. For this virus, the formation of double membrane vesicles where replication occurs relies on LC3 and intersects with ER-associated degradation (ERAD) machinery [111], illustrating that viruses can utilize autophagy proteins and structures that resemble autophagic vesicles independently of the conventional autophagy pathway.

## 7. Conclusions and Future Directions

In summary, the interrelationship between the autophagy machinery and HCV has emerged as a highly active research area and there is now little doubt that HCV utilizes autophagy proteins for its propagation [41–45]. It is also clear that vesicles with characteristic features of autophagic vacuoles accumulate in HCV infected cells [41–44,46,68]. Importantly, consistent with the ability of HCV to induce autophagy in infected cells *in vitro* and supportive of the physiological and clinical relevance of the *in vitro* studies discussed in this review, Rautou *et al*. recently reported evidence for an increased autophagic response in the liver of chronically HCV infected patients relative to the normal liver and to patients with nonalcoholic steatohepatitis, alcoholic liver disease, and chronic hepatitis B infection [121]. Mechanism(s) are beginning to emerge to explain how HCV regulates the autophagy pathway [44,46] and how it exploits autophagy proteins to initiate replication of the incoming viral genome [41,43] and to attenuate RIG-I signaling [44,45]. Further work will be necessary to reconcile our fragmented and sometimes conflicting understanding of the role of autophagy in HCV infection. In particular, much remains to be learned about the spatio-temporal appearance and composition of vesicles resembling autophagosomes in HCV-infected cells. Autophagy has long been considered as a random cytoplasmic degradation process, but it is now clear that autophagosomes can degrade substrates in a selective manner. In particular, ubiquitin and/or specific adaptor proteins could provide selectivity for degradation by autophagy [122,123]. As we learn (i) the identity of membrane donors from which autophagy vesicles are formed in HCV infected cells, (ii) the nature of the cellular organelles and/or protein aggregates engulfed in these vesicles, and (iii) the extent to which they resemble or differ from canonical autophagic vesicles, we will better understand how autophagy proteins can regulate HCV replication and propagation.

Finally, the recent demonstration that pharmacologic modulation of autophagy augments the efficacy of currently available anticancer regimens [124] and the possibility that autophagy may be a legitimate target for the development of antimicrobial drugs [125,126] are very exciting areas in biomedical research. It is conceivable that inhibition of autolysosome maturation may, directly or indirectly via increased RIG-I signaling, repress HCV propagation. Nonetheless, it is important to recognize that our current understanding of the role of the autophagy machinery in the propagation and control of virus infections is in its infancy, and much more work must be done before we can confidently translate these results into new approaches for the treatment of this serious and widespread human disease.
